# Medical Symbolism Illustrated

**Published:** 1891-10

**Authors:** 


MEDICAL SYMBOLISM ILLUSTRATED. By Thomas S. Sozinskey, M. D., Ph. D: F. A. Davis, Publishers, Philadelphia and London.

This little work is erudite and instructive to a superlative degree. It comprises much that is necessary and much that is classical.

Few of the profession possess the time, energy or inclination to hunt, for themselves, what Dr. Sozinskey so compactly and succinctly presents, and for these, especially, will this little volume prove valuable. H. H.

